# Development of a Real-Time TaqMan RT-PCR Assay for the Detection of NADC34-like Porcine Reproductive and Respiratory Syndrome Virus

**DOI:** 10.3390/vetsci10040279

**Published:** 2023-04-06

**Authors:** Teng Tu, Maonan Pang, Dike Jiang, You Zhou, Xulong Wu, Xueping Yao, Yan Luo, Zexiao Yang, Meishen Ren, Aiping Lu, Ge Zhang, Yuanyuan Yu, Yin Wang

**Affiliations:** 1 Key Laboratory of Animal Diseases and Human Health of Sichuan Province, College of Veterinary Medicine, Sichuan Agricultural University, Chengdu 611130, China; 2019303092@stu.sicau.edu.cn (T.T.); 2020203022@stu.sicau.edu.cn (M.P.); 2020103005@stu.sicau.edu.cn (D.J.); 2021203043@stu.sicau.edu.cn (Y.Z.); 13577@sicau.edu.cn (X.Y.); 41187@sicau.edu.cn (Y.L.); 13643@sicau.edu.cn (Z.Y.); 14773@sicau.edu.cn (M.R.); 2 Chengdu Agricultural College, Chengdu 611130, China; yaanwuxl@163.com; 3 Law Sau Fai Institute for Advancing Translational Medicine in Bone and Joint Diseases (TMBJ), School of Chinese Medicine, Hong Kong Baptist University, Hong Kong SAR, China; aipinglu@hkbu.edu.hk (A.L.); zhangge@hkbu.edu.hk (G.Z.); yuyuanyuan@hkbu.edu.hk (Y.Y.); 4 Guangdong-Hong Kong-Macao Greater Bay Area International Research Platform for Aptamer-Based Translational Medicine and Drug Discovery (HKAP), Hong Kong SAR, China; 5 Institute of Integrated Bioinformedicine and Translational Science (IBTS), School of Chinese Medicine, Hong Kong Baptist University, Hong Kong SAR, China

**Keywords:** NADC34-like porcine reproductive and respiratory syndrome virus, real-time TaqMan RT-PCR, detection method

## Abstract

**Simple Summary:**

It is vitally important that scientists are able to describe their work simply and concisely to the public, especially in an open-access online journal. The simple summary consists of no more than 200 words in one paragraph and contains a clear statement of the problem addressed, the aims and objectives, pertinent results, conclusions from the study, and how they will be valuable to society. This should be written for a lay audience, i.e., no technical terms without explanations. No references are cited, and no abbreviations. Submissions without a simple summary will be returned directly.

**Abstract:**

NADC34-like porcine reproductive and respiratory syndrome virus first appeared in 2017 in a herd of pigs in Liaoning Province, China. The virus was subsequently found in other provinces. Given the potential for this virus to cause an epidemic, rapid, sensitive, and specific detection of NADC34-like PRRSV is required. The virus’ *ORF5* gene was artificially synthesized based on a Chinese reference strain, and specific primers/probes for the *ORF5* gene were designed. Then, the amplified target fragment was cloned into the pMD19-T vector, and a series of diluted recombinant plasmids were used to generate a standard curve. An optimized real-time TaqMan RT-PCR method was established. The method was highly specific for NADC34-like PRRSV, without cross-reactions with other non-targeted pig viruses. The detection limit of this assay was 10^1^ copies/μL. The method had an efficiency of 98.8%, a squared regression value (R^2^) of 0.999, and showed a linear range of 10^3^–10^8^ copies/μL of DNA per reaction. This method was shown to be analytically specific and sensitive with a low intra- and inter-assay coefficient of variation (<1.40%). A total of 321 clinical samples were tested using the established method, and four were shown to be positive (1.24%). This study confirmed the existence of NADC34-like PRRSV and HP-PRRSV co-infection in Sichuan and provided a promising alternative tool for the rapid detection of NADC34-like PRRSV.

## 1. Introduction

Porcine reproductive and respiratory syndrome (PRRS) is the main cause of reproductive disorders in pregnant sows and of respiratory symptoms in piglets. The main symptoms of PRRSV infection in sows are depression, loss of appetite or abandonment, fever, breathing difficulties of different degrees, and abortion in late gestation. Piglets show typical respiratory symptoms, dyspnea, sometimes abdominal breathing, loss of appetite or abandonment, an increase in body temperature to more than 40 °C, and, in a small number of piglets, purple skin on the ears and body can be observed [1,2,3]. The pathogen PRRSV is a member of the Arteriviridae family in the order Nidovirales [4]. Its RNA is a single plus strand, and its genome length is about 15 kb, containing at least 10 open reading frames (ORFs) [5,6,7]. The ORF1a encodes the non-structural proteins NSP1α, NSP1β, NSP2-NSP6, NSP7α, NSP7β and Nsp8, and ORF1b encodes the non-structural proteins Nsp9, Nsp10, Nsp11, and Nsp12. *ORF2a*, *ORF2b*, *ORF3-ORF5*, *ORF5a*, *ORF6*, and *ORF7* encode structural proteins GP2a, E, GP3, GP4, GP5, GP5a, M, and N, respectively [8,9,10]. Among them, ORF5 (GP5) is the main protective antigenic protein of PRRSV. Therefore, the *ORF5* gene is often used as the basis for genotyping in the analysis of molecular genetic variation of the virus [11,12,13,14]. In addition, *ORF5* is considered to be the most variable gene of PRRSV among PRRSV genotypes [15,16,17]. According to Pirzadeh’s study, the amino acid sequence similarity of *ORF5* between different genotypes of PRRSV was 52~55% [18]. Therefore, *ORF5* can be used as a target gene to establish detection methods to distinguish different genotypes of PRRSV.

According to the differences in genome sequence and antigenic characteristics, PRRSV can be divided into two different species: PRRSV-2 (represented by the VR-2332 strain) and PRRSV-1 (represented by the Lelystad virus strain) [19]. According to the phylogenetic analysis of the PRRSV *ORF5* gene, PRRSV-2 can be divided into nine lineages, among which five have been reported in China. They include harmonics 1 (sublineage 1.5, NADC30 PRRSV and sublineage 1.8, NADC34 PRRSV), 3, 5 (sublineage 5.1), 8 (sublineage 8.7), and 9. In the global PRRSV classification system, Lineage 1 PRRSV occupies about 4900 samples (37%), which is a very large cladogram, and these strains are mainly distributed in the United States, Canada, and China [20].

NADC34-like PRRSV was first reported in the United States in 2014 and has been detected in more than five states in recent years [21]. In 2017, two NADC34-like PRRSV strains (LNWK96 and LNWK130) were detected for the first time in Liaoning Province, China [22]; these strains were named NADC34 PRRSV because they had 100 consecutive aa deletion characteristics in the Nsp2 gene compared with the VR2332 strain and were highly homologous to the IA/2014/NADC34 PRRSV strain in the United States [23]. In 2022, NADC34-like PRRSV strains (SCcd2020) were detected for the first time in Sichuan Province, China [24]. In recent years, the number of NADC34-like PRRSV strains in China and its distribution provinces has increased. However, there have been no relevant reports of NADC34-like PRRSV detection in other regions. Therefore, in order to detect and distinguish NADC34-like PRRSV from other PRRSV strains in a timely manner, an efficient, sensitive, and rapid detection method is needed to provide specific diagnosis during the epidemic of NADC34-like PRRSV in China so as to conduct accurate clinical investigations in the shortest time.

## 2. Materials and Methods

### 2.1. Strains and Clinical Samples

All viral strains used in this study were collected in our laboratories, including PRRSV, HP-PRRSV, PRRSV NADC30-like, porcine epidemic diarrhea virus, classical swine fever virus attenuated vaccine, porcine parvovirus, and pseudorabies virus. A total of 321 samples (201 blood, 30 lung tissues, 60 saliva swabs, and 30 semen samples) were investigated from 15 pig farms located in Sichuan Province, China (Guangyuan, Chengdu, Guangan, Suining, Yibing, and Nanchong) between 2018 and 2022 and submitted to the College of Veterinary Medicine, Sichuan Agricultural University. All 321 samples were collected from pigs suspected of being infected with PRRSV from 15 pig farms in Sichuan Province that reported PRRSV outbreaks from 2018 to 2022.

### 2.2. DNA/RNA Extraction Method

In this study, we used the TaKaRa DNA/RNA extraction kit for the DNA/RNA extraction of tissues and viral strains. The DNA/RNA extraction kit and reverse transcription kit were purchased from TaKaRa (Beijing, China).

### 2.3. Construction of the ORF5 Gene Plasmid

The *ORF5* gene of NADC34-like PRRSV (603 bp) was synthesized artificially by Sangon Biotech (Shanghai, China) based on a reference sequence of a Chinese isolate available in GenBank (Accession number: MH370474.1) and cloned into pUC57 Vector by Sangon Biotech. The pUC57-ORF5 plasmid was obtained from Sangon Biotech, and the plasmid DNA was amplified in *Escherichia coli* DH5α, subsequently purified using the plasmid MiniPrep Kit and quantified using a NanoDrop 2000 spectrophotometer.

### 2.4. Primers and Probe for NADC34-like PRRSV ORF5 Gene

The genome sequences of Classical-PRRSV (Accession number: AF066183.4, AF159149.1, AY150564.1, AY585241.1, FJ175688.1), HP-PRRSV (Accession number: EF112445.1, EU236259.1, KP771777.1, EU825724.1, FJ797690.1), NADC30 PRRSV(Accession number: MH500776.1, MF375260.1, JN654459.1, KY373214.1, MW880772.1), NADC34-like PRRSV (Accession number: MF326985.1, MF326995.1, MF326997.1, MF326994.1, MK453050.1, OL771208.1, OL516358.1, OL771209.1, OL771207.1, KT257967.1) strains/isolates were retrieved from the GenBank and aligned using the software program DNAStar (DNASTAR, Madison, WI, USA). Since the *ORF5* gene was highly conserved within the NADC34-like PRRSV and showed the highest diversity between Classical-PRRSV, HP-PRRSV, and NADC30 PRRSV, it was selected as the molecular target for the real-time PCR. The primers and probe sets were selected using AlleleID (version 6.0) (Beijing Tianyan Rongzhi Software Co., Ltd., Beijing, China). The forward primer was N34-F (5′-CCTGTGTTGACTCATATTGTCTCC-3′); and the TaqMan probe was N34-P (FAM-5′-CGCCCTCACCACCAGCCATTTCCT-3′-BHQ1); the reverse primer was N34-R (5′-CGGCGTAAATGCTACTCAAGAC-3′). The length of the amplicon was 130 bp.

### 2.5. Cloning and Sequencing

Plasmid pUC57-ORF5 was used as a template for amplification using the N34-F/R primer set. The amplified target fragment was cloned into the pMD-19T Simple vector (TaKaRa, Dalian, China) for DH5α (TIANGEN) transformation. After colony screening and identification, positive clones were grown in a 5 mL LB culture medium, and plasmid isolation was performed using a plasmid MiniPrep Kit (Sangon Biotech). DNA from the plasmid was sent to Sangon Biotechnology for sequencing. The concentration of plasmid pMD19T-N34 was determined using a NanoDrop 2000 spectrophotometer (Thermo Fisher Scientific, Waltham, MA, USA).

### 2.6. Optimization of the Real-Time RT-PCR

A real-time RT-PCR was performed on a CFX Connect Real-Time PCR Detection System (Bio-Rad, Hercules, CA, USA) in a reaction volume of 20 μL containing 10 μL of SsoAdvanced Universal Probe Supermix (Bio-Rad), different volumes of each primer (10 μmol/L) and probe (10 μmol/L), 2 μL of DNA template, and RNase-free deionized distilled water (ddH_2_O). RT-qPCR amplification conditions: 2 min initial denaturation at 94 °C, 40 cycles at 94 °C for 10 s, and final extension at 55.4 °C for 30 s.

### 2.7. Method Standardization

Ten-fold serial dilutions of pMD19T-N34 plasmid were performed. The plasmids were detected three times by RT-qPCR in the range from 7.80 × 10^9^ copies/μL to 7.80 × 10^4^ copies/μL. The standard curve of Ct versus plasmid copies was then constructed by CFX Manager (Bio-Rad).

### 2.8. Specificity Analysis

Specificity amplification was performed using the TaqMan RT-PCR method established in this study. Briefly, 10 ng of viral DNA or cDNA from PRRSV, HP-PRRSV, PRRSV, NADC30-like, PEDV, CSFV, PPV, and PRV were used as templates for amplification by real-time PCR. ddH_2_O was used as a negative control, and pMD19T-N34 plasmid was used as a positive control. Each test was performed in triplicate.

### 2.9. Sensitivity Analysis

The pMD19-N34 with a concentration of 10^8^ to 10^0^ copies/μL was used to evaluate the sensitivity of the real-time RT-PCR. The sensitivity of RT-qPCR was evaluated using the optimal reaction conditions with the minimum detection amount in the RT-qPCR method established in this study.

### 2.10. Repeatability and Reproducibility Analyses

To analyze the reproducibility and repeatability of the real-time RT-PCR, 10^5^, 10^4^, 10^3^, and 10^2^ copies of pMD19-N34 were tested in triplicate in three independent experiments. The inter-assay variability was evaluated in three independent runs and in different thermocyclers (Roche Diagnostics, Risch-Rotkreuz, Switzerland; ABI, Forster, CA, USA; Bio-Rad, USA) performed on different days. Ct values for each dilution in each trial were used to calculate the mean, standard deviation, and coefficient of variation (CV). The range (minimum and maximum values) for each parameter was determined. SPSS statistical software (IBM, Armonk, NY, USA) was used to calculate the mean, standard deviation, and CV.

### 2.11. Detection of NADC34-like PRRSV in Clinical Samples

Viral RNA was extracted from 321 clinical samples and reversely transcribed into cDNA, then amplified using the developed real-time RT-PCR method. NADC34-like PRRSV positive determination: samples amplified before 38 cycles and with a typical amplification curve. If a typical magnification curve with a Ct value greater than 38 was observed, the evaluation was repeated. Highly pathogenic PRRSV and NADC30-like PRRSV were also detected in the above samples by referring to the previous RT-qPCR method [25]. Table 1 provides information on the RT-qPCR methods used to detect all PRRSV strains.

## 3. Results

### 3.1. Optimization Results of Real-Time RT-qPCR System

The 20 μL reaction system consisting of 10 μL SsoAdvanced Universal Probe Supermix (Bio-Rad), 2 μL DNA template, 5.8 μL RNase-ddH_2_O free, 1 μL of each primer (8 μmol/L), and 0.2 μL of solution of probe (10 μmol/L) was found to produce the strongest fluorescent signals. By using the pUC57-ORF5 plasmid as a template, the target fragment of 130 bp was successfully amplified with specific primers. The PCR product was cloned into the pMD19-T vector to construct pMD19T-N34 recombinant plasmid (GenBank accession number OP716633). DNAMAN (LynnonBiosoft, San Ramon, CA, USA) was used to compare the DNA sequencing results for the isolates from the samples with the reference sequence and revealed no mutation in the sequence of the DNA insert, and the consistency was 100%, showing that the pMD19T-N34 recombinant plasmid standard had been successfully constructed. The plasmid concentration was 7.80 × 10^9^ copies/μL.

### 3.2. Standard Curve

**Figure 1:**
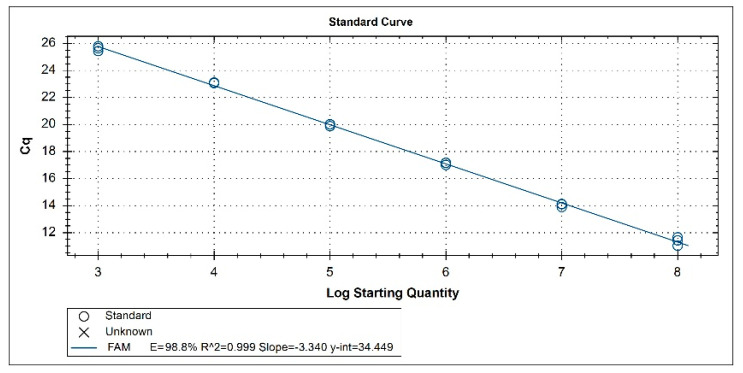
Standard curve of NADC34-like PRRSV real-time RT-PCR for serially diluted. The standard curve represents three replicates of each plasmid concentration (between 7.80 × 10^8^ copies/μL and 7.80 × 10^3^ copies/μL). Ct values from three replicates (*y*-axis) are plotted versus logarithmic concentrations of plasmid copies (*x*-axis).

pMD19T-N34 recombinant plasmid was diluted tenfold in RNase-free ddH_2_O, and the diluted samples were used as the template for the real-time RT-PCR. As shown in Figure 1, the generated standard curve had a good linear correlation between 7.80 × 10^8^ copies/μL and 7.80 × 10^3^ copies/μL (slope: −3.340; y-int: 34.449; correlation coefficient R^2^: 0.999; amplification efficiency: 98.8%).

### 3.3. Specificity Analysis

**Figure 2:**
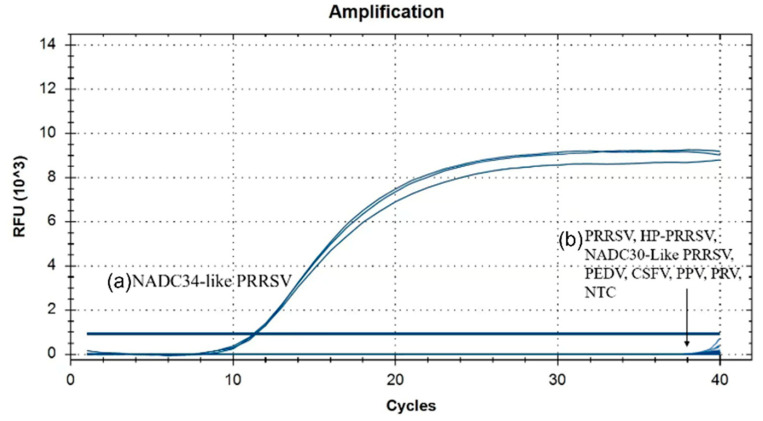
Specificity of NADC34-like PRRSV gene based on the qRT-PCR assay. (**a**) Represented the real-time RT-PCR amplification curve of NADC34-like PRRSV plasmid; (**b**) Represented the real-time RT-PCR amplification results of PRRSV, PRRSV, HP-PRRSV, NADC30-like PRRSV, PEDV, CSFV, PPV, PRV, and no-template control (NTC).

Using the viral DNA and cDNA as templates, the results (Figure 2) showed that only pMD19T-N34 plasmid (NADC34-like PRRSV) was detected by the real-time RT-PCR, and other viruses were not detected. Similar results were obtained from three repeated independent reactions, demonstrating that the assay was highly specific.

### 3.4. Sensitivity Analysis

**Figure 3:**
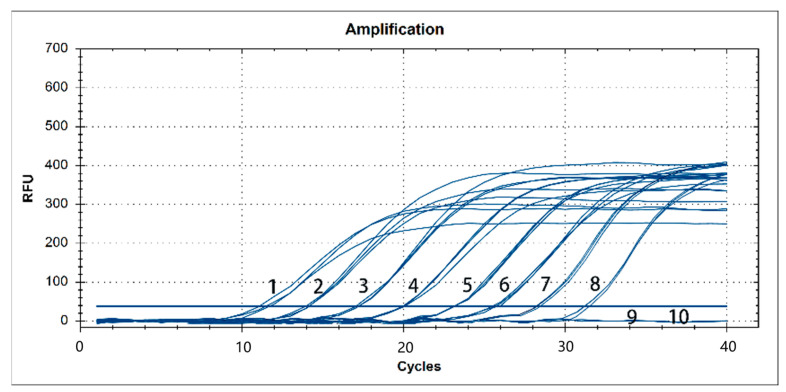
Sensitivity analysis of the TaqMan real-time RT-PCR for NADC34-like PRRSV. RFU: relative fluorescence units; 1–9: 7.80 × 10^8^ copies/μL–7.80 × 10^0^ copies/μL; 10: negative control(ddH_2_O). The lowest copy number detected by RT-qPCR was 7.80 × 10^1^ copies/μL.

A dilution range of 10^8^–10^0^ copies/μL of pMD19-T-N34 as a template was used. Our results showed that the real-time PCR had a detection limit of 10^1^ copies, and similar results were obtained from three repeated independent reactions (Figure 3).

### 3.5. Reproducibility Analysis

In order to evaluate the repeatability of the detection method, the assay was carried out using recombinant plasmids at three different concentrations from 7.80 × 10^5^ to 7.80 × 10^2^ copies/μL. Each concentration was evaluated three times, and the determined Ct values, mean, standard deviation, and CV are shown in Table 2. Our results showed that the experimentally established method for the detection of NADC34-like PRRSV by fluorescence quantitative RT-PCR has good reproducibility. Therefore, this method can be used for the stable and reliable detection of NADC34-like PRRSV from the samples.

### 3.6. Detection of NADC34-like PRRSV in the Clinical Samples

A total of 321 samples from pig farms in different areas of Sichuan Province from 2018 to 2022 were used to evaluate the practicability of a TaqMan-based quantitative RT-PCR (RT-qPCR) assay. Part of the collected samples is shown in Figure S1. The Ct values and virus copies of positive clinical specimens are shown in Table S1. In Table 3, the positive rate of NADC34-like PRRSV was 1.24% (4/321). For the NADC30-like PRRSV, 11 samples were positive, and the positive ratio was 9.35% (30/321). For the HP-PRRSV, 77 samples were positive, and the positive ratio was 23.99% (77/321). The co-infection ratio of NADC30-like PRRSV and HP-PRRSV was 7.48% (24/321). The co-infection ratio of NADC34-like PRRSV and HP-PRRSV was 0.62% (2/321).

## 4. Discussion

Although the onset time, genome structure, and clinical symptoms of PRRSV-1 and PRRSV-2 are similar, the consistency of their whole genome sequences is only about 60% [26]. PRRSV-2 strains were mainly prevalent in China before 2012, including isolate CH-1A (1996, belonging to CH-1A-like PRRSV) [27], isolate BJ-4 (2000, belonging to VR2332-like PRRSV) [28], isolates JXA1 and HUN4 (2006, belonging to HP-PRRSV) [29], and isolates QYYZ and GM2 (2010, belonging to QYYZ-like PRRSV) [30]. Since 2012, NADC30-like PRRSV has gradually become popular in China, and it is easy to recombine with other subtypes of PRRSV. NADC34-like PRRSV isolates (LNWK96, LNWK130) first appeared in the Liaoning Province of China in 2017 [22]. Later, the existence of NADC34-like PRRSV was reported in Fujian Province (isolate FJ0908) [31] and Heilongjiang Province (isolate HLJDZD32-1901) [32]. In May 2022, NADC34-like PRRSV isolates (SCcd2020) was also reported in Sichuan Province [24].

Since the epidemic of NADC30-like PRRSV, several studies have reported the detection methods of NADC30-like PRRSV and PCR detection methods that can differentiate and diagnose different subtypes of PRRSV. Li designed a pair of conventional primers according to the number of amino acids missing in the Nsp2 gene of three NADC30-like PRRSV, HP-PRRSV, and CA-PRRSV and established an RT-PCR method to identify different subtypes of PRRSV [33]. However, compared with NADC34-like PRRSV, NADC30-like PRRSV only has a few discontinuous aa deletion features in the Nsp2 gene, and the difference is not obvious, so it is impossible to accurately determine the position changes through gel electrophoresis. Nanhua Chen [34] successfully established a four-fold real-time PCR typing method for PRRSV1, NADC30-like PRRSV, HP-PRRSV, and CA-PRRSV. However, the primers and probes designed to diagnose NADC30-like PRRSV are targeted at the ORF6 gene, which is well conserved. NADC34-like PRRSV and NADC30-like PRRSV have high homology on this gene, so they cannot be distinguished. For NADC34-like PRRSV, these detection methods cannot be used for direct differential diagnosis and can only be determined by gene sequencing and sequence homology comparison. Therefore, it is very important to establish a rapid and effective nucleic acid diagnostic method to detect the clinical prevalence of NAD34-like PRRSV, which can effectively reduce the risk of missing the detection of Nad34-like PRRSV strains.

Real-time fluorescent PCR is widely used in clinical detection. Compared with SYBR Green-based PCR, the advantage of real-time TaqMan PCR is that it yields highly specific, low false positives and read-through signals generated by the TaqMan probe. Compared with conventional PCR, real-time PCR is able to detect the reaction process in real time and determine the absolute number of amplified products, with strong specificity and better sensitivity. The developed real-time PCR assay is rapid, with no requirement for post-PCR steps. In this study, the two-step PCR method was used to establish the real-time TaqMan RT-PCR method. Compared with one-step PCR, the disadvantage of the two-step method is that the operation steps are slower and more complex, which will increase the probability of contamination and the mismatch rate of the PCR reaction. However, the advantage of the two-step method is that the intermediate cDNA is easy to preserve, which is of great significance for the repeated detection of a large number of samples. In addition, only 1/10 of the retro-transcriptional reaction products were taken for the reaction in the second PCR step, and the remaining reaction products were conducive to the adjustment of PCR conditions with strong reproducibility.

The clinical sample study showed that NADC34-like PRRSV did not appear in these 15 pig farms in Sichuan Province before 2022. However, four cases of NADC34-like PRRSV were detected in 2022, two of which were co-infected with HP-PRRSV, suggesting that the emergence of NADC34-like PRRSV may be related to the recombination of NADC34-like PRRSV and HP-PRRSV. At the same time, we found that the NADC30-like PRRSV and HP-PRRSV co-infection rates increased year by year, and the most prevalent PRRSV in Sichuan Province from 2018 to 2022 was HP-PRRSV (with a positive rate from 17.74% to 29.82%).

Since the emergence of PRRSV in China, PRRS has caused serious harm to the country’s swine industry. Since 2012, HP-PRRSV has been spotted as the main circulating strain. However, co-circulation of new mutant strains, i.e., NADC30-like, GM2, and NADC34-like, have been reported in pig farms from some areas across China [35,36]. According to a survey [37], the HP-PRRSV strain was found to be the most prevalent (51.57%) PRRSV strain circulating in China between the years 2017 to 2018, which was consistent with the results of the PRRSV survey in this study. Zhou’s study [38] collected 231 samples from different pig farms in four provinces in Eastern China from 2017 to 2022; he found that the prevalent PRRSV strain in Eastern China was still HP-PRRSV, while the proportion of NADC30-like and NADC34-like strains have increased, which is consistent with the results in our study, the positive rate of NADC30-like PRRSV increased from 3.23% to 12.36% between 2018 and 2022. A study [39] investigated the positive rate of PRRSV in South China from 2017 to 2021 and found that the detection rate of PRRSV in South China showed an increasing trend year by year from 2017 to 2021, and PRRSV in South China was still dominated by highly pathogenic strains, which was consistent with the clinical test results in our study. However, they found that NADC30-like strains were the other main type circulating in South China from 2017 to 2021 and exhibited a yearly decreasing detection rate. On the contrary, in our study, the positive rate of NADC30-like PRRSV in Sichuan Province showed an increasing trend, indicating that the outbreak of NADC30-like PRRSV had obvious regional differences. The increased detection rate of NADC30-like PRRSV in Sichuan may be related to the recombination events, which usually occur between different PRRSV isolates and are considered to be one of the most important mechanisms of PRRSV emergence and evolution [40,41].

At present, NADC34-like PRRSV is only detected in China sporadically, and so far, there has been no large-scale outbreak, which is similar to the local prevalence of NADC30-like PRRSV in the early stage. If NADC34-like PRRSV is as widely prevalent as NADC30-like PRRSV in China, a rapid and accurate detection method is needed. In this study, we successfully developed and evaluated a real-time RT-PCR assay for the rapid and accurate detection of NADC34-like PRRSV. The real-time TaqMan RT-PCR method established in this study had an efficiency of 98.8%, a squared regression value (R^2^) of 0.999, and showed a linear range of 10^3^–10^8^ copies of DNA per reaction. The method detected down to a lower limit of 10^1^ copies, and it was analytically specific and sensitive with low intra- and inter-assay CV (<1.40%). The experimental results show that the real-time TaqMan RT-PCR method established in this study is reliable and has good reproducibility. It can provide rapid and accurate results for the detection of NADC34-like PRRSV, which is of great significance for monitoring this virus.

## Figures and Tables

**Table 1:** Information on the RT-qPCR methods.

Type of Virus	Primers and Probe(5′-3′)	Target Gene
NADC34-like PRRSV	F: CCTGTGTGACTCATATTGTCTCC R: CGGCGTAAATGCTACTCAAGAC P: FAM-CGCCCTCACCACCAGCCATTTCCT-BHQ1	*ORF5*
HP- PRRSV	F: GACGTGCCCCCAAGCTGAT	*Nsp2*
	R: GGATGCCCATGTTCTGCGA P: FAM-CGTAGAACTGTGACAACAACGCTGAC-BHQ1	
NADC30-like-PRRSV	F: CGTATTGGACACCTCTTTTGACTG R: AACTGGACCTAATCTTCCTGCG P: ROX-CCCAAAGGTCTTCGTCGGTATTCC-BHQ2	*Nsp2*

**Table 2:** Repeatability test of RT-qPCR.

Positive Plasmid Concentration	Intra-Assay		Inter-Assay	
(copies/µL)	Ct (Mean ± SD)	CV (%)	Ct (Mean ± SD)	CV (%)
7.80 × 10^5^	19.98 ± 0.11	0.55	20.04 ± 0.15	0.75
7.80 × 10^4^	23.09 ± 0.13	0.56	23.11 ± 0.21	0.91
7.80 × 10^3^	25.65 ± 0.21	0.82	25.80 ± 0.36	1.40
7.80 × 10^2^	28.23 ± 0.25	0.89	28.45 ± 0.31	1.09

**Table 3:** Detection results of NADC34-like PRRSV in the archived clinical samples from 2018 to 2022 by real-time RT- PCR.

Year	Sample Number	NADC34-like PRRSV		NADC30-like PRRSV		HP-PRRSV		NADC34-like PRRSV + NADC30-like PRRSV		NADC30-like PRRSV + HP-PRRSV		NADC34-like PRRSV + HP-PRRSV	
		Number	Ratio	Number	Ratio	Number	Ratio	Number	Ratio	Number	Ratio	Number	Ratio
2018	62	0	0	2	3.23%	11	17.74%	0	0	1	1.61%	0	0
2019	53	0	0	5	9.43%	14	26.42%	0	0	3	5.66%	0	0
2020	60	0	0	7	11.67%	17	28.33%	0	0	5	8.33%	0	0
2021	57	0	0	5	8.77%	14	24.56%	0	0	5	8.77%	0	0
2022	89	4	4.49%	11	12.36%	21	23.60%	0	0	10	11.24%	2	2.25%
Total	321	4	1.24%	30	9.35%	77	23.99%	0	0	24	7.48%	2	0.62%

## Data Availability

All data generated or analyzed in this study are presented within the tables and figures of the manuscript. The datasets (RNA sequencing data, GenBank accession number OP716633) generated and/or analyzed during the current study are available in the NCBI (https://www.ncbi.nlm.nih.gov/nuccore/MH370474.1 (accessed on 23 January 2023)).

## Supplementary Materials

The following supporting information can be downloaded at: https://www.mdpi.com/article/10.3390/vetsci10040279/s1, Figure S1: Several samples were collected with suspected PRRSV infection; Table S1: Ct values and viral copies of positive clinical samples.
