# Supplementary material for: Development of a Real-Time TaqMan RT-PCR Assay for the Detection of NADC34-like Porcine Reproductive and Respiratory Syndrome Virus
Source: Vet Sci. 2023 Apr 6;10(4):279. doi: 10.3390/vetsci10040279 (PMC10141196; doi:10.3390/vetsci10040279)
Supplement: Supplementary file 1 [file vetsci-10-00279-s001.zip › vetsci-2207831-SI.pdf]

Supplementary Materials

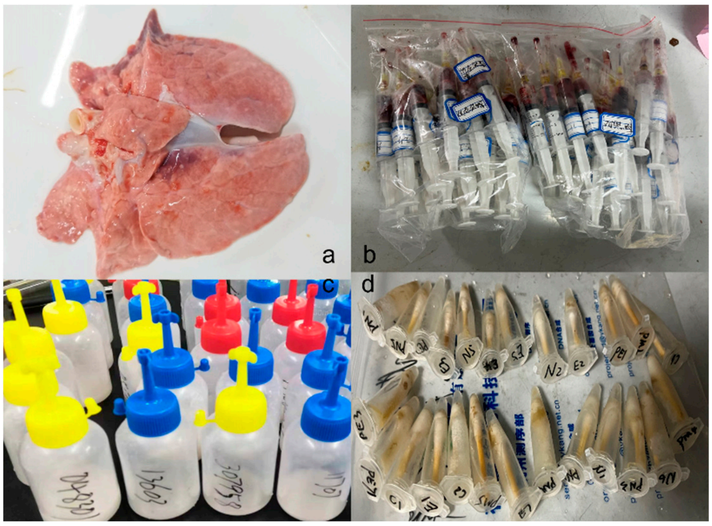

Figure S1. Several samples were collected with suspected PRRSV infection; (a) Collected pig's lung (b) Collected pig's blood (c) Collected pig essence (d) Collected saliva swabs

Table S1. Ct values and viral copies of positive clinical samples.

Year	Positive samples	Ct values	viral copies	Co-infected samples
2018	N30-1	30.21	1125	
	N30-2	28.13	4022	
	HP-1	19.74	35862067	
	HP-2	18.46	78814659	
	HP-3	24.61	1792833	
	HP-4	27.89	238362	
	HP-5	31	35185	
	HP-6	25.19	1254827	
	HP-7	32.13	17557	N30-1/HP-3
	HP-8	17.49	143138814	
	HP-9	15.88	385383989	
	HP-10	16.98	195889770	
	HP-11	21.22	14428812	
2019	N30-1	31.29	580	
	N30-2	19.74	686108	
	N30-3	16.86	4004551	
	N30-4	27.63	5463	
	N30-5	24.44	38553	
	HP-1	25.49	20264	
	HP-2	18.52	75958622	
	HP-3	18.47	78331302	
	HP-4	19.39	44477790	
	HP-5	22.99	4856773	
	HP-6	34.01	5523	
	HP-7	23.84	2879104	
	HP-8	21.68	10872627	N30-1/HP-10,N30-4/HP-10,N30-3/HP-6
	HP-9	31.06	33910	
	HP-10	14.01	1217567144	
	HP-11	26.58	533610	
	HP-12	29.47	90181	
	HP-13	16.77	222903293	
	HP-14	27.98	225524	
2020	N30-1	21.64	214259	
	N30-2	24.79	31114	
	N30-3	33.41	158	
	N30-4	33.25	175	

	N30-5	19.81	657311	
	N30-6	19.93	610727	
	N30-7	26.66	9896	N30-2/HP-11,N30-5/HP-10,N30-4/HP-7,N30-6/HP-4,N30-7/HP-8
	HP-1	17.53	139659602	
	HP-2	17.98	105887911	
	HP-3	19.01	56190907	
	HP-4	24.37	2078067	
	HP-5	20.09	28915282	
	HP-6	19.21	49685878	
	HP-7	19.56	40061304	
	HP-8	24.12	2423544	
	HP-9	35.01	2986	
	HP-10	30.41	50580	
	HP-11	25.47	1056277	
	HP-12	26.73	486575	
	HP-13	21.33	13484734	
	HP-14	26	762395	
	HP-15	23.14	4428667	
	HP-16	29.43	92428	
	HP-17	16.55	255207167	
2021	N30-1	19.41	839812	
	N30-2	22.83	103363	
	N30-3	33.12	189	
	N30-4	30.36	1026	
	N30-5	16.32	5574541	
	HP-1	16.47	268081038	
	HP-2	21.01	16418569	
	HP-3	19.66	37671121	
	HP-4	15.36	530664045	
	HP-5	14.52	889689733	
	HP-6	16.32	293995649	
	HP-7	15.71	427869946	
	HP-8	15.99	360168292	N30-1/HP-6,N30-2/HP-10,N30-3/HP-11,N30-4/HP-3,N30-5/HP-8
	HP-9	17.32	158918892	
	HP-10	17.85	114703819	
	HP-11	16.76	224278756	
	HP-12	19.36	45306255	
	HP-13	26.35	614713	
	HP-14	21.36	13238153	
	N30-1	19.31	892863	
	N30-2	18.66	1329545	
	N30-3	19.98	592306	
	N30-4	21.34	257482	
	N30-5	30.06	1233	
	N30-6	30.41	995	
	N30-7	18.72	1281567	
	N30-8	21.39	249716	
	N30-9	31.14	636	
	N30-10	19.34	876605	
2022	N30-11	19	1079577	
	HP-1	18.06	100802929	
	HP-2	14.32	1006170671	N30-1/HP-4,N30-2/HP-6,N30-3/HP-7,N30-4/HP-3,N30-5/HP-10,N30-7/HP-12,N30-8/HP-13,N30-9/HP-15,N30-10/HP-16,N30-11/HP-19,N34-2/HP-21,N34-2/HP-1
	HP-3	19.36	45306255	
	HP-4	15.62	452227185	
	HP-5	16.32	293995649	
	HP-6	17.91	110547253	
	HP-7	21.39	12996082	
	HP-8	19.34	45867120	
	HP-9	20.68	20114066	
	HP-10	24.63	1770911	

HP-11	14.96	678712446
HP-12	22.74	5664208
HP-13	30.58	45558
HP-14	16.98	195889770
HP-15	17.83	116123786
HP-16	29.37	95903
HP-17	17.93	109195477
HP-18	19.6	39087552
HP-19	18.4	81778082
HP-20	19.81	34350554
HP-21	21.03	16217802
N34-1	19.68	26415
N34-2	18.2	73277
N34-3	20.32	16992
N34-4	16.74	200491

Note: N34:NADC34-like-PRRSV; N30:NADC30-like-PRRSV; HP:HP-PRRSV. The conversion of Ct values and virus copies of HP PRRSV, and NADC30-like-PRRSV was referred to the reference [21].
